# New Copy Number Variations in Schizophrenia

**DOI:** 10.1371/journal.pone.0013422

**Published:** 2010-10-13

**Authors:** Chiara Magri, Emilio Sacchetti, Michele Traversa, Paolo Valsecchi, Rita Gardella, Cristian Bonvicini, Alessandra Minelli, Massimo Gennarelli, Sergio Barlati

**Affiliations:** 1 Division of Biology and Genetics, Department of Biomedical Sciences and Biotechnology, Brescia University School of Medicine, Brescia, Italy; 2 Department of Mental Health, Brescia Spedali Civili, Brescia, Italy; 3 University Psychiatric Unit, Brescia University School of Medicine, Brescia, Italy; 4 Centre on Behavioural and Neurodegenerative Disorders, Brescia University and EULO, Brescia, Italy; 5 Genetics Unit, IRCCS San Giovanni di Dio, Fatebenefratelli, Brescia, Italy; University of Queensland, Australia

## Abstract

Genome-wide screenings for copy number variations (CNVs) in patients with schizophrenia have demonstrated the presence of several CNVs that increase the risk of developing the disease and a growing number of large rare CNVs; the contribution of these rare CNVs to schizophrenia remains unknown. Using Affymetrix 6.0 arrays, we undertook a systematic search for CNVs in 172 patients with schizophrenia and 160 healthy controls, all of Italian origin, with the aim of confirming previously identified loci and identifying novel schizophrenia susceptibility genes. We found five patients with a CNV occurring in one of the regions most convincingly implicated as risk factors for schizophrenia: NRXN1 and the 16p13.1 regions were found to be deleted in single patients and 15q11.2 in 2 patients, whereas the 15q13.3 region was duplicated in one patient. Furthermore, we found three distinct patients with CNVs in 2q12.2, 3q29 and 17p12 loci, respectively. These loci were previously reported to be deleted or duplicated in patients with schizophrenia but were never formally associated with the disease. We found 5 large CNVs (>900 kb) in 4q32, 5q14.3, 8q23.3, 11q25 and 17q12 in five different patients that could include some new candidate schizophrenia susceptibility genes. In conclusion, the identification of previously reported CNVs and of new, rare, large CNVs further supports a model of schizophrenia that includes the effect of multiple, rare, highly penetrant variants.

## Introduction

In recent years, the availability of high throughput technologies investigating the genome at a resolution intermediate between that of cytogenetic analysis (>2–5 Mb) and DNA sequencing (1–700 bp) led to the demonstration that a large number of genomic sequences, many of which encompass entire genes, vary in copy number among individuals. These intermediate size deletions and duplications, referred to as copy number variations (CNVs), are more common in the general population than ever imagined before and could account for more genomic differences among sindividuals than single nucleotide polymorphisms (SNPs) [1], [2]. Recent microarray studies also identified many CNVs in a variety of complex mental disorders such as mental retardation [3], [4], autism spectrum disorders [5]–[8], and schizophrenia [9], [10].

Regarding schizophrenia, genome-wide screening for CNVs has demonstrated that deletions and duplications that disrupt genes are more common in patients than in healthy subjects [11], [12]. The pathogenicity of these CNVs seems to be correlated with their size, because patient–control differences have involved mainly large copy number variants [11], [13], [14]. Some of the large CNVs were observed in several patients with schizophrenia [11], [13]–[20]; others were described in only one or very few patients [12]–[14], [16], [21]. In the case of the rare, large CNVs, however, their contribution to the disorder cannot be ruled out. The observation that deletions greater than 2 Mb occur extremely rarely, less than 0.04%, in healthy, cognitively unimpaired individuals seems indeed to justify the inference that their presence, even in a single individual, could have a high prior probability of being associated with disease [14]. Furthermore, some of these CNVs were found to be associated with a broad range of neuropsychiatric phenotypes crossing the traditional boundaries of diagnosis [9].

Thus, these studies point strongly to a model of schizophrenia pathogenesis that includes the effects of many different structural variants. However, the fact that the CNVs identified so far occurred at a combined frequency of only 2–3%, leaves still undiscovered the vast majority of information on susceptibility to schizophrenia [9].

In order to search for novel schizophrenia susceptibility genes and/or loci for those reported in previous studies, and integrate the databases of the CNVs putatively related to schizophrenia susceptibility, we undertook a further systematic search for CNVs in patients with schizophrenia and healthy controls, both of Italian origin.

## Results

### Overall CNVs

Of the 180 patients with schizophrenia and 171 healthy controls who were analyzed with the Affymetrix 6.0 microarrays, 172 cases and 160 controls survived the filtering for quality control and population stratification. A total of 4193 autosomal CNVs larger than 100 kb, called by at least 25 probes with an average distance lower than 10 kb, were identified; 2189 were among the patients and 2004 were in the control group (Table 1). The list of these CNVs is available as a supporting file (File S1) uploadable in the UCSC Genome Browser, (http://genome.ucsc.edu/).

**Table 1:** Distribution of CNVs in patients and controls^a^.

	No. of Subjects	No. of CNVs	CNVs/person	t-test p-value	No. of Deletions	No. of Duplications	Deletions/Duplications	χ^2^ p-value
Controls	160	2004	12.5±3.7	0.31	656	1348	0.49	0.09
Patients	172	2189	12.7±3.7		771	1418	0.54	
Total	332	4193	—	—	1427	2766	—	—

a Only autosomal CNVs greater than 100 kb, called by at least 25 probes with an average distance between markers of less than 10 kb have been considered.

The overall CNV rate per person was not statistically different (t-test, p = 0.31) between patients (12.7±3.7 SD) and controls (12.5±3.7 SD). The deletion/duplication ratio was similar in the 2 sample populations (cases, 0.54; controls, 0.49; χ^2^, p = 0.09) (Table 1).

The median dimension of the CNVs was 204 and 209 kb for the patients and controls, respectively. The largest CNV, 2566 kb, was found in a patient; the largest CNV among the controls was 1837 kb.

### Rare CNVs

Among the 4193 CNVs found, 179 can be considered rare. Among the 179 CNVs, 85 were found in the controls (CNV/person = 0.53±0.84) and 94 in the patients (CNV/person = 0.55±0.83). The deletions (N = 70) occurred at a modest, but significantly higher frequency (χ^2^, p = 0.026) in patients (46.8%) compared with controls (30.6%). This is because the deletion/duplication ratio significantly increased in patients when only rare CNVs were considered (from 0.54 to 0.88; χ^2^, p = 0.022). This was not the case for controls (from 0.49 to 0.44; χ^2^, p = 0.679).

Most of the rare CNVs described in patients occurred as singletons and only 12 were observed in more than one case; of these 12 CNVs, 10 involved both patients and controls and the remaining 2 were found in more than one patient but never in controls. These 2 rare CNVs common to patients were a duplication mapping at 3q21.2 (126.9–127.1 Mb) not encompassing any gene, and a deletion mapping at 10q23.33 (96.4–96.5 Mb) completely deleting a copy of the *CYP2C18* gene and part of one predicted isoform of the flanking gene, *CYP2C19*.

**Figure 1:**
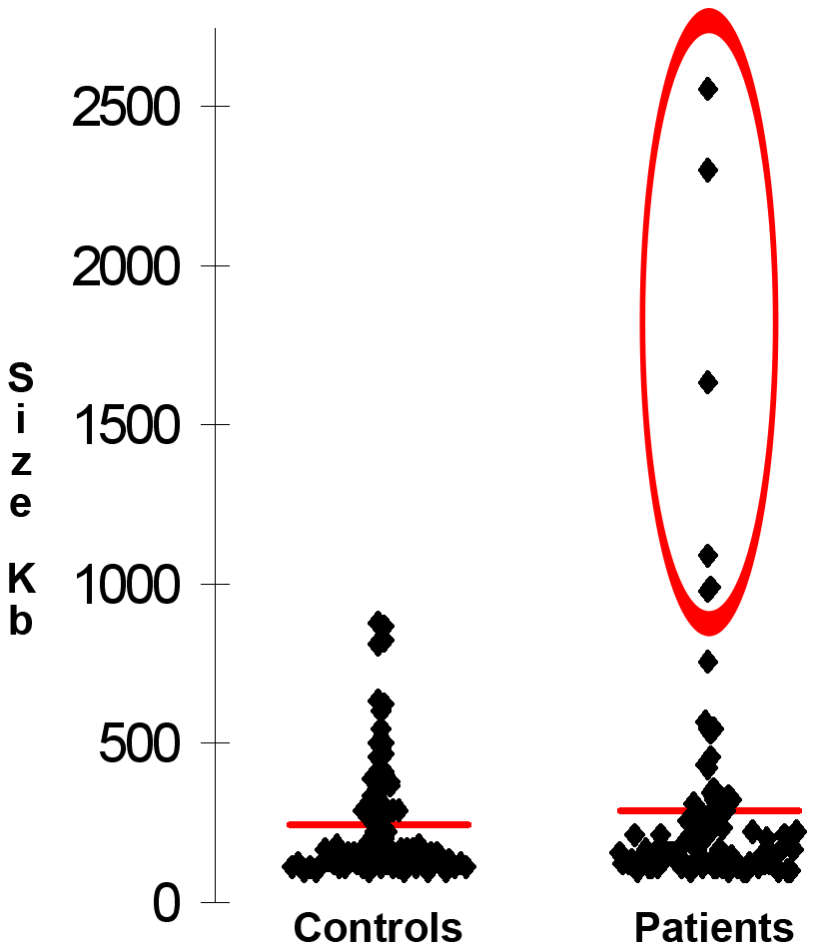
Scatter plot of rare CNV size. Scatter plot of the distribution of rare CNVs as a function of their dimension in controls and patients. CNVs larger than 900 kb are circled in red.

Regarding the size, rare CNVs larger than 900 kb did not occur in the control group but were observed in 6 patients (Figure 1). All these CNVs encompassed at least one gene and the deletion/duplication ratio was 5∶1 in favour of the deletions. The list of these large CNVs is reported in Table 2 together with the overlapping loci previously implicated in schizophrenia. The duplication on chromosome 17 has been reported previously in a healthy control [13], but the deletions on chromosomes 2, 4, 5, 8 and 11 are reported for the first time.

**Table 2:** List of all relevant CNVs found in our samples.

State	Chr	Cytoband	Start^a^	End	Size (kb)	Loss/Gain	No. Of Markers	Genes
Case	2	p16.3	50,952,424	51,280,162	328	Loss	229	*NRXN1*
Case	2	q12.2–q12.3	106,232,909	107,873,857	1641	Loss	1013	*PLGLA*, *RGPD3*, *ST6GAL2*, *RGPD4*
Case	3	q29	197,185,549	198,838,373	1653	Loss	829	*TFRC*, *ZDHHC19*, *OSTalpha*, *PCYT1A*, *TCTEX1D2*, *TM4SF19*, *UBXN7*, *RNF168*, *C3orf43*, *WDR53*, *FBXO45*, *LRRC33*, *C3orf34*, *PIGX*, *PAK2*, *SENP5*, *NCBP2*, *LOC152217*, *PIGZ*, *MFI2*, *DLG1*, *BDH1*, *LOC220729*
Case	4	q32.1–q32.2	160,885,459	163,187,767	2302	Loss	1583	*FSTL5*
Case	5	q14.3	83,318,004	84,407,494	1089	Loss	593	*EDIL3*
Case	8	q23.3	112,965,276	115,531,385	2566	Loss	1417	*CSMD3*
Case	11	q25	133,461,668	134,449,982	988	Loss	749	*JAM3*, *NCAPD3*, *VPS26B*, *THYN1*, *ACAD8*, *GLB1L3*, *GLB1L2*, *B3GAT1*
Case	15	q11.2	18,700,540	20,968,714	2268	Loss	629	*GOLGA6L6*, *GOLGA8C*, *BCL8*, *LOC646214*, *CXADRP2*, *POTEB*, *NF1P1*, *LOC727924*, *OR4M2*, *OR4N4*, *OR4N3P*, *GOLGA8DP*, *GOLGA6L1*, *TUBGCP5*, *CYFIP1*, *NIPA2*, *NIPA1*, *WHAMML1*, *GOLGA9P*, *HERC2P2*
Case	15	q11.2	20,224,752	20,777,909	553	Loss	284	*GOLGA6L1*, *TUBGCP5*, *CYFIP1*, *NIPA2*, *NIPA1*, *WHAMML1*
Case	15	q13.2-q13.3	28,280,641	30,702,885	2422	Gain	1160	*DKFZP434L187*, *CHRFAM7A*, *FAM7A1*, *FAM7A2*, *ARHGAP11B*, *MTMR15*, *MTMR10*, *TRPM1*, *MIR211*, *KLF13*, *OTUD7A*, *CHRNA7*, *FAM7A1*, *FAM7A2*, *ARHGAP11A*
Case	16	p13.11	15,435,743	16,189,809	754	Gain	494	*C16orf45*, *KIAA0430*, *NDE1*, *MIR484*, *MYH11*, *C16orf63*, *ABCC1*, *ABCC6*
Case	17	p12	14,039,002	15,414,023	1375	Loss	1109	*COX10*, *CDRT15*, *HS3ST3B1*, *MGC12916*, *CDRT7*, *PMP22*, *TEKT3*, *CDRT4*, *FAM18B2*
Case	17	q12	28,983,508	29,962,135	979	Gain	933	*ACCN1*, *CCL* genes, *MCP3*, *TMEM132E*

a Genome coordinates refer to the NCBI36/hg18 assembly.

### Identification of CNVs already reported to be associated with schizophrenia

In relation to the CNVs most convincingly implicated in the risk for schizophrenia [9], [10], [22], we found a patient with a deletion of 328 kb erasing the 5′UTR and the first 3 exons of the *NRXN1* gene (NM_004801.4) and two patients with a deletion in the 15q11.2 region. Moreover duplications at 15q13.3 and at 16p13.11 were found in single patients (Table 2). None of these CNVs were found in controls. No patient presented CNVs at the 1q21, 16p11.2 and 22q11.1 loci. For CNVs previously described only in patients with schizophrenia [11]–[14] but with limited evidence of association with the disorder, deletions in 2q12.2 (106.2–107.9 Mb), 3q29 (197.2–198.8 Mb) and 17p12 (14.0–15.4 Mb) were found in single patients (Table 2).

## Discussion

This CNV analysis adds further weight to recent proposals that high penetrant deletions may account, especially when rare, for a portion of genetic susceptibility to schizophrenia [11], [13], [14], but does not confirm previous reports [11], [12] that people with schizophrenia have a generalized increase in all the CNVs combined. The study also contributes to the growing list of specific CNVs potentially implicated in schizophrenia. Among the CNVs more typically associated with schizophrenia, those located in the *NRXN1* gene and at the 15q11.2, 15q13.3 and 16p13.11 loci collectively were found in 3% of our sample, a figure close to prevalence rates already reported [9]. The 15q11.2 deletion was found only in two patients (1%); this is in agreement with the frequency reported by other groups in patients with schizophrenia (0.55–0.85%) [11], [13], [19].

Deletions in the region of the 15q13.3 locus are generally considered pathogenic and have been found to be associated with a broad spectrum of phenotypes ranging from schizophrenia to mental retardation and autism [9]; the reciprocal duplications, although less frequent, have already been observed at higher frequency in patients with schizophrenia [11], [13], [14]. This also suggests that the 15q13.3 duplication may be a risk factor for schizophrenia [9], [14].

**Figure 2:**
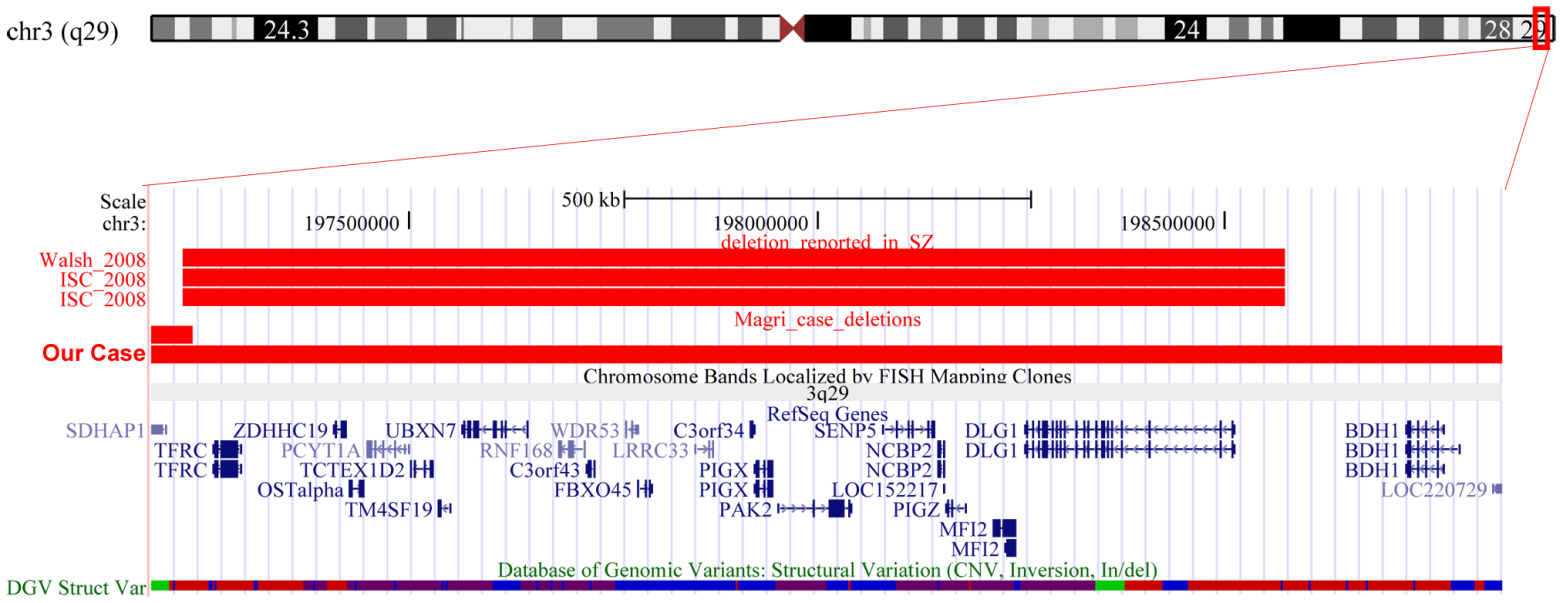
CNVs at 3q29. UCSC Genome Browser image of the 3q29 region found deleted in a patient with schizophrenia from this study and in three patients from previous studies [11], [12].

**Figure 3:**
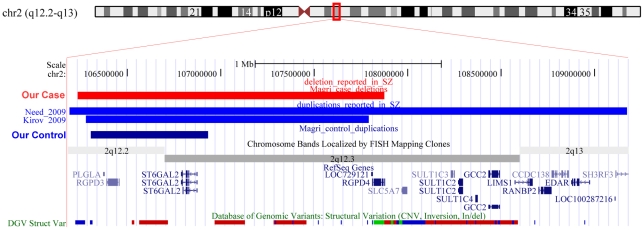
CNVs at 2q12. UCSC Genome Browser image of the 2q12 region found deleted in a patient and duplicated in a control in our study. The same region was previously reported as duplicated in two other patients with schizophrenia [13], [14]. Deletions and duplications are shown in red and blue, respectively.

Among CNV candidates for an association with schizophrenia but not yet replicated, the current genome-wide screening study has found 3 deletions. One is a large deletion at 17p12 that overlapped the copy number state region found associated with schizophrenia by Kirov et al. [13] and involving the *peripheral myelin protein 22* gene (*PMP22*). The second is a deletion in 3q29 that overlapped the CNV observed initially by Walsh et al. [12] in 1 of 150 patients with schizophrenia and subsequently by the International Schizophrenia Consortium (ISC) in 2 of 3391 cases [11] (Figure 2). Combining previous and current data, it follows therefore that the 3q29 deletion has so far been observed in 0.1% of patients (4 out of 3719) but not in 3609 healthy controls. The 3q29 deleted region included at least 20 genes and, among these, the most promising seems to be the *Discs Large homolog 1* (*DLG1*) which encodes for the presynaptic protein SAP97. Furthermore, and more specifically, a recent study [23] has found an association between some SNPs inside the *DLG1* gene and males with schizophrenia. The third CNV is a deletion at the 2q12.2 region that erases 4 genes. This deletion has been reported for the first time in this study, but duplications of the same region have already been reported [13], [14] in single patients with schizophrenia and have been observed in one of our healthy control (Figure 3). So far, CNVs in 2q12 have been reported to occur almost 10 times more frequently (0.18% vs 0.02%) in people with schizophrenia (3 of 1656 cases) than in healthy controls (1 of 4036 individuals). Prevalence rates on CNVs at 2q12.2 therefore suggest that it could represent a schizophrenia susceptibility risk factor, but further data are required before concluding that this region has a role in schizophrenia pathogenesis.

The present study also suggests a possible involvement in schizophrenia of new genes or loci inside large (>900 kb) regions deleted only in patients. This is in agreement with proposals of previous studies [13], [14] that very large CNVs might have a high prior probability of being associated with schizophrenia phenotypes when observed almost exclusively in patients with schizophrenia. One of the new deleted genes identified, *follistatin-like 5* (*FSTL5*) located in the 4q32.1–32.2 region, encodes for the follistatin-related protein 5 precursor, an extracellular protein mainly expressed in B lymphoblasts, cerebellum and cerebellum peduncles, and possibly involved [24] in the early development of chemoattraction events responsible for the projection of the axons of the dorsal root ganglion neurons toward the dorsal spinal cord. A further new deletion, at 5q14.3, involved the *EGF-like repeats and Discoidin I-Like domains 3* (*EDIL3*) gene, which encodes an integrin-ligand promoting adhesion of endothelial cells, inhibits formation of vascular-like structures and might be involved in regulation of vascular morphogenesis of remodelling during embryonic development [25].

However, the *CUB* and *Sushi multiple domains 3* (*CSMD3*) and *beta-1,3-glucuronyltransferase 1* (*B3GAT1*) genes located in the 8q23.3 and 11q25 regions, respectively, probably constitute the 2 new, most promising deleted genes. *CSMD3* is one of the 3 members of the *CSMD* gene family that encodes for proteins with CUB and sushi multiple domains. The *CSMD3* gene is weakly expressed in most tissues except the adult and fetal brain [26], [27]. A recent study [28] proposed *CSMD3* as a candidate gene for autistic spectrum disorders, because it maps in a break point region common to 2 unrelated autistic patients carrying balanced chromosome translocations t(5;8)(q14.3;q23.3) and t(6;8)(q13;q23.2), respectively. In addition, *CSMD3* maps in a locus found to be linked to benign adult familial myoclonic epilepsy [27]. These data suggest that *CSMD3* could be a potential susceptibility candidate gene not only for schizophrenia, but for a broader range of neuropsychiatric disorders. *B3GAT1*, a member of the glucuronyltransferase gene family, encodes for a protein that is the key enzyme during the biosynthesis of the carbohydrate epitope HNK-1, present on a number of cell adhesion molecules important for neurodevelopment. Knockout mice for the *B3GAT1* orthologous gene exhibit impaired synaptic plasticity and spatial learning [29], 2 abnormalities also described in patients with schizophrenia [30]. Furthermore, Jeffries and colleagues [31] described a family with the chromosome translocation t(6;11)(q14.2;q25) segregating with schizophrenia-like phenotype; because the breakpoint of chromosome 11 is close to the *B3GAT1* gene, it has been hypothesized [31] that polymorphic or other variations of the 11q telomere might affect the activity of *B3GAT1*, thus becoming a risk factor for schizophrenia and related psychoses.

In summary, the identification of new, rare, large CNVs spanning potential candidate schizophrenia susceptibility genes, as well as the CNVs previously reported to be associated with schizophrenia, supports once again a model of schizophrenia that includes among its causes the effect of many, rare, highly penetrant structural variants. However, the limited number of patients (<10%) with a potential pathologic CNV suggests that these structural variations, even if they constitute a risk factor for schizophrenia phenotype, are not the only risk factor.

## Materials and Methods

### Subjects

The study involved 351 subjects, 180 patients with schizophrenia and 171 healthy controls.

In agreement with our other recent reports [32], [33], to be eligible, both patients and controls had to be white and of Italian descent for at least 2 generations, have no relatives among other prospective participants, have given written informed consent, and fulfil predefined group-specific inclusion and exclusion criteria.

Patients had to satisfy the DSM-IV-TR criteria [34] for schizophrenia in the absence, during their lifespan, of co-morbidities with other DSM-IV-TR Axis I disorders, with the exception of nicotine and caffeine abuse.

The controls had to have no lifetime personal evidence of any DSM-IV-TR Axis I disorder, again with the exception of nicotine and caffeine abuse, and to present a negative family history for psychoses and mood disorders in their first-degree relatives.

The diagnosis of schizophrenia was made following a detailed clinical interview, complemented by a revision of all medical records and, when required, a DSM-IV-TR adjusted version of the Standardized Clinical Interview for DSM-IV Axis I Disorders, Clinician Version [35]. The healthy controls were initially asked about previous specialist examinations and consumption of psychopharmacological agents exceeding a sporadic use of benzodiazepines and hypnotics for tension and/or insomnia. Prospective controls who gave negative answers to these 2 questions also underwent detailed clinical interviews complemented, if necessary, by a DSM-IV-TR adjusted version of the Diagnostic Interview for Genetic Studies [36].To exclude a family history for psychoses and mood disorders in healthy controls, they and, in case of doubt, a family member, were interviewed following an ad hoc questionnaire.

A team of psychiatrists with a common experience working on patients with schizophrenia was in charge of clinical assessments and decisions about eligibility for the study, after dedicated training on the procedures and demonstration of valid inter-rater reliability. Two physicians in the team independently evaluated each prospective participant. In cases of discordance, a joint revision of all the material took place in the presence of an independent referee who made the final decision after discussion.

The patients were enrolled from those admitted to facilities of the Brescia University and Spedali Civili Psychiatric Unit and the Brescia IRCSS Fatebenefratelli. The controls consisted of doctors, nurses, employers and attendants working in the 2 hospitals, students of Brescia University, or their relatives.

The format of the written informed consent was approved by a local Ethic Committee (CEIOC) (act number 3/2004 of 01/22/2004). The consent form provided a concise but unequivocal explanation about the aims of the study and an explicit guarantee of anonymity and the impossibility of identification details as a unique number linked all the individual data.

### Genotyping

All samples were genotyped by Affymetrix Human Mapping GeneChip 6.0 arrays with a total of 2 millions of probes, half of which were polymorphic. DNA was processed according to the instructions provided in the Affymetrix Genome-Wide Human SNP *Nsp*/*Sty* 6.0 Assay Manual. Initial analysis of the array to obtain intensity data was performed using Affymetrix GeneChip Command Console Software (AGCC). The AGCC probe cell intensity data were then analyzed with GenotypeConsole 3.01 (GTC3.01) to obtain genotype data. Quality thresholds were used to reduce the number of genotype errors. According to Affymetrix specifications, samples that had a QC≤0.4 were discarded from further analyses. After this quality step, 3 samples were excluded from population structure analysis and from copy number state analysis.

### Quality controls of population structure

The chp.files generated by the Birdseed v2 algorithm implemented in GTC 3.01 were imported into the HelixTree 7.0.3 software for statistical analyses (Golden Helix SVS, Bozeman, MT, USA). Before performing any type of analysis, cleaning of the data was performed. In particular, from the initial 906,599 SNPs, we excluded from the analysis those SNPs on the sex chromosomes, those with a call rate <95%, a minor allele frequency <0.1 and those with Hardy–Weinberg disequilibrium p-values <1×10^–7^. After these steps, 560,044 SNPs were retained for population stratification analysis.

Population stratification, which can be responsible for spurious associations, was controlled by means of an enhanced version of EIGENTRAT present in HelixTree 7.0.3. Using this approach, we identified 26 outlier samples. The analysis of these samples with GenotypeColour™ [37] revealed the presence of cryptic relatedness among the samples. After taking the relatedness into account, 13 samples were discarded from further analysis. The re-analysis of the data after the elimination of related samples colocated the previous outlier samples with all the others, confirming that cryptic relatedness and not other types of stratification was the cause of their previous marginal position.

### Generation of CNVs calls

All subjects who passed the QC threshold and population stratification procedures were used for copy number (CN) state analysis. The CN state calls were generated from the BRLMM-P-Plus algorithm implemented in GTC 3.0.1. This algorithm compared the intensity signal of each marker in each sample against a reference pool formed from a group of 270 samples derived from the HapMap database. After this comparison, the software generates a median intensity value for each marker, and the value obtained is reported as the log2 ratio or CN state. According to Affymetrix protocols, samples with a median absolute pairwise difference (MAPD) ≥0.4 were discarded from further analyses. After this quality control step, 3 samples were eliminated. The copy number state information of the final sample of 332 subjects (172 patients with schizophrenia and 160 healthy controls) was then used by the Affymetrix segmentation algorithm to identify CNVs. To reduce the presence of false-positive CNVs, the segmentation algorithm parameters were set to consider as a CNV only those regions larger than 100 kb, comprised of at least 25 contiguous markers without diploid state and with an average probe density lower than 10 kb. The segmentation algorithm was also used to extrapolate from all the CNVs identified only those with at least 50% overlap with CNV regions previously reported by other authors in patients with schizophrenia [12]–[14], [16], [17], [19]–[21].

### Statistical analysis on CNV calls

The t-test was used to evaluate patient–control differences in the average number of CNVs per sample, whereas the χ^2^-test was used to compare the frequency distribution of deletions and duplications in the 2 populations. The one-tailed was used to test if CNVs stratified by size or type (duplication or deletion) were significantly associated with schizophrenia.

### Analysis of rare CNVs

To verify the presence of rare, relatively highly penetrant CNVs that could be responsible for a proportion of the schizophrenia phenotype, we defined as rare those CNVs with a ≤80% overlapping segment with a CNV previously described in the DGV (release 18v6; http://projects.tcag.ca/variation/). The choice of this threshold is arbitrary, but substantially the same results were obtained if only those CNVs with 0% overlap were retained or only those with 100% overlap were discarded (data not shown).

### Validation of CNVs larger than 900 kb

Validation of CNVs larger than 900 kb was performed by real-time polymerase chain reaction technology. Applied Biosystems TaqMan® Copy Number Assays Hs06710043_cn, Hs06722515_cn, Hs05233869_cn, Hs00492735_cn were used to validate CNVs inside the region 4q32.1-32.2, 5q14.3, 11q25 and 17q12, respectively. Custom TaqMan® Copy Number Assays ST6GAL2_cn_1 and CSMD3_cn_1 were used to validate CNVs inside the 2q12.2-q12.3 and 8q23.3 regions, respectively. Assays were performed in quadruplicate in 96-well plates with 5 ng of DNA per reaction (for the custom assays,10 ng were used). Copy numbers were determined using CopyCaller software v.1.0 (Applied Biosystems).

### Note Added in Proof

While this paper was under review, Mulle et al. (Am. J. Hum. Genet 87, 229–236) reported that “Microdeletions of 3q29 Confer High Risk for Schizophrenia”. These data confirm our hypothesis of the relevance of deletions in this region in schizophrenia pathogenesis.

## Supporting Information

File S1 The list of CNVs identified in this study. A total of 4193 autosomal CNVs larger than 100 kb, called by at least 25 probes with an average distance lower than 10 kb.
(0.13 MB TXT)
